# Single-cell profiling of physically interacting immune cells using image-guided sorting

**DOI:** 10.64898/2026.10.09.758094

**Published:** 2026-10-16

**Authors:** Ningxin Kang, Cheryl Kim, Thomas J Scriba, Bjoern Peters, Julie G Burel

## Abstract

Single-cell transcriptomics of cell-cell complexes offers key insights into *in vivo* immune interactions, yet distinguishing true physical contacts from artifacts remains challenging. Here, we integrated spectral imaging cell sorting with physical cell deconvolution to separate circulating T cell-monocyte doublets from tuberculosis patients into physically interacting (in-contact) and non-interacting (coincidental) populations. Single-cell sequencing of individual cell partners revealed that physical contact is associated with robust signatures distinct from singlets, including unidirectional monocyte-to-T cell RNA transfer, heightened metabolic activity and T cell cytotoxicity, and a trend toward antigen-specific enrichment. These signatures spanned all T cell and monocyte subpopulations, reflecting a global program of T cell-monocyte engagement. Coincidental doublets displayed intermediate transcriptional profiles between singlet and in-contact states, suggesting the presence of recently disrupted or forming physical pairs. This framework provides high-fidelity deconvolution of cellular interactions, uncovering signatures governing T cell-monocyte communication in human disease.

